# Recombination Events Involving the *atp9* Gene Are Associated with Male Sterility of CMS PET2 in Sunflower

**DOI:** 10.3390/ijms19030806

**Published:** 2018-03-11

**Authors:** Antje Reddemann, Renate Horn

**Affiliations:** Institut für Biowissenschaften, Abt. Pflanzengenetik, Universität Rostock, Albert-Einstein-Straße 3, D-18059 Rostock, Germany; antje.reddemann@web.de

**Keywords:** *atp9*, cytoplasmic male sterility, CMS PET1, CMS PET2, *Helianthus annuus*, plant mitochondria, recombination, RNA-editing, respiration

## Abstract

Cytoplasmic male sterility (CMS) systems represent ideal mutants to study the role of mitochondria in pollen development. In sunflower, CMS PET2 also has the potential to become an alternative CMS source for commercial sunflower hybrid breeding. CMS PET2 originates from an interspecific cross of *H. petiolaris* and *H. annuus* as CMS PET1, but results in a different CMS mechanism. Southern analyses revealed differences for *atp6*, *atp9* and *cob* between CMS PET2, CMS PET1 and the male-fertile line HA89. A second identical copy of *atp6* was present on an additional CMS PET2-specific fragment. In addition, the *atp9* gene was duplicated. However, this duplication was followed by an insertion of 271 bp of unknown origin in the 5′ coding region of the *atp9* gene in CMS PET2, which led to the creation of two unique open reading frames *orf288* and *orf231*. The first 53 bp of *orf288* are identical to the 5′ end of *atp9*. *Orf231* consists apart from the first 3 bp, being part of the 271-bp-insertion, of the last 228 bp of *atp9*. These CMS PET2-specific orfs are co-transcribed. All 11 editing sites of the *atp9* gene present in *orf231* are fully edited. The anther-specific reduction of the co-transcript in fertility-restored hybrids supports the involvement in male-sterility based on CMS PET2.

## 1. Introduction

Cytoplasmic male sterility (CMS) is a maternally inherited incapability of higher plants to produce or shed functional pollen [1]. CMS has been described for more than 150 plant species [2,3], and is often associated with mitochondrial rearrangements and the expression of new open reading frames (orfs) leading to the translation of unique proteins that appear to interfere with mitochondrial functions and pollen development [4]. Exploitation of CMS systems is the most cost-effective way to produce hybrids. Hybrid production is widely used in field crops to gain enhanced yield and yield stability by using heterosis effects [5]. Hybrid breeding based on a CMS-system most frequently consists of a three line system: the CMS line, which is maintained by an isonuclear maintainer line present on a normal fertile cytoplasm, and a restorer line carrying one or two dominant nuclear restorer-of-fertility (*Rf*) genes to restore male fertility in the F1-hybrids. These restorer genes interact with the mitochondrial transcripts to suppress the deleterious effect of CMS by diverse mechanisms and thereby allow the production of male-fertile F1-hybrids [1,6].

In sunflower, CMS PET1 is so far the only cytoplasm worldwide used for the commercial hybrid production [7]. CMS PET1 was originally derived from the interspecific cross of *H. petiolaris* Nutt. × *H. annuus* [8] and has been used the last 50 years in sunflower hybrid breeding [9]. This reduction to one CMS source carries the risk of a high vulnerability of the cytoplasm to pathogen attacks, as the interaction of *Bipolaris maydis* with the T-cytoplasm in maize has demonstrated [10,11]. However, more than 70 CMS sources have been reported in the FAO Technical Consultation of the European Cooperative Research Network on Sunflower [12]. These CMS sources have either occurred spontaneously in wild populations or were derived from interspecific crosses or mutagenesis experiments. Prerequisite of the utilization of one of these alternative CMS sources in hybrid breeding is their molecular characterization and the identification of suitable restorer lines. This has happened so far only for very few of these alternative CMS sources [13,14,15,16,17,18]. In sunflower, the most comprehensive study of 28 male sterile cytoplasms and the fertile, normal cytoplasm was based on hybridization patterns obtained with different mitochondrial genes, which grouped the CMS sources and the fertile cytoplasms into 10 classes using the UPGMA (Unweighted Pair Group Method with Arithmetic Mean) method [19,20].

**Figure 1:**
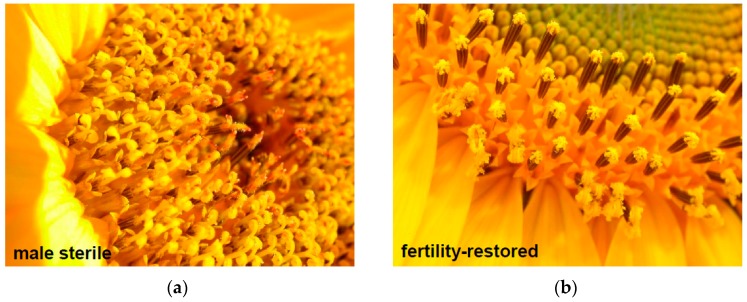
Inflorescence in the present of the CMS PET2 in a maintainer and restorer background. (**a**) CMS PET2 × RHA265, male sterile; (**b**) CMS PET2 × IH-51, fertility-restored hybrid.

In this study, CMS PET2, an alternative CMS source derived from an interspecific cross *Helianthus petiolaris* Nutt. × *Helianthus annuus* L. [21], was analyzed. Whereas CMS PET1 went together with nine PET1-like CMS sources [22] into group MT-θ, CMS PET2 grouped together with CMS GIG1 (*H. giganteus* L. × *H. annuus* L., [23]) into MT-γ [19]. Although CMS PET1 and CMS PET2 have the same parental origin regarding the involved species, the differences in the restriction fragment patterns between the mtDNAs of CMS PET1 and CMS PET2 indicate a different molecular mechanism behind male sterility in these two CMS cytoplasms [19]. In CMS PET1, pollen development is aborted in the tetrad stage of meiosis II due to premature programmed cell death of the tapetum cells initiated by the release of cytochrome C from the mitochondria [24,25]. Only very rudimentary very small anthers are formed in CMS PET1, but sunflower lines carrying the PET2 male-sterile cytoplasm still form medium sized anthers (Figure 1). For CMS PET1, it is known that reorganization of mtDNA generated a new open reading frame *orfH522* (coding for a protein of about 16 kDa), which is co-transcribed with *atpA*, now called atp1, and is responsible for the male-sterile phenotype in sunflower CMS PET1 [26,27,28]. Anther-specific reduction of the co-transcript of *atp1* and *orfH522* restores male fertility [29,30]. Heterologous expression of *orfH522* in tapetal cell layers of tobacco induced male sterility [31], whereas RNAi-mediated silencing of *orfH522* restored fertility [32]. For CMS PET2 and CMS GIG1, a CMS-specific protein of 12.4 kDa was identified by *in organello* translation [15]. However, the corresponding open reading frame has not yet been identified.

In recent years, mitochondrial DNAs have been sequenced to answer several questions [33,34,35,36,37]. Some mitochondrial genome sequences have been successfully used to identify CMS-specific open reading frames [38,39,40], whereas in other studies no clear answers regarding the CMS mechanism were obtained like in wheat [41] or in pigeonpea [42]. However, only the fertile mitochondrial genome with 300,945 bp [43] and the mtDNAs of CMS PET1 (MG735191.1) have been sequenced and assembled in sunflower, but not CMS PET2.

To investigate the cause of aberrant pollen development in CMS PET2, we analyzed the organization of the mitochondrial DNA, the occurrence of specific new open reading frames, their transcription profiles and their absence in CMS PET1 and the male-fertile line HA89 as reference. The occurrence of two novel open reading frames, *orf288* and *orf231,* inside a second copy of the *atp9* gene and their possible role in causing male-sterility in CMS PET2 are discussed. In addition, diagnostic markers for CMS PET1, CMS PET2 and the fertile cytoplasm in sunflower are presented that can be applied in hybrid breeding.

## 2. Results

### 2.1. Identification of Recombination Events in CMS PET2

**Figure 2:**
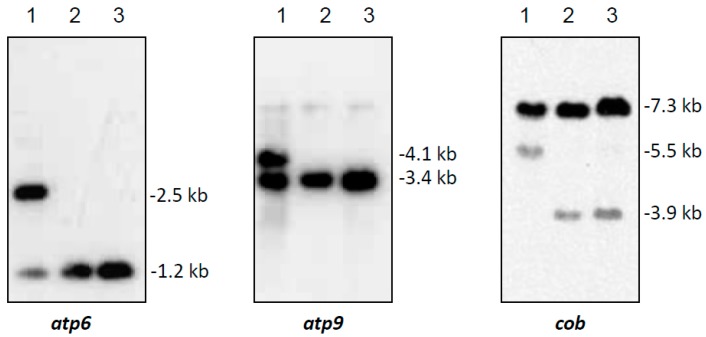
Southern hybridization pattern of mtDNA digested with *Hind*III using the mitochondrial genes *atp6*, *atp9* and *cob* as probes. Lanes: 1, CMS PET2 (male sterile); 2, CMS PET1; 3, HA89 (fertile, normal cytoplasm).

**Figure 3:**
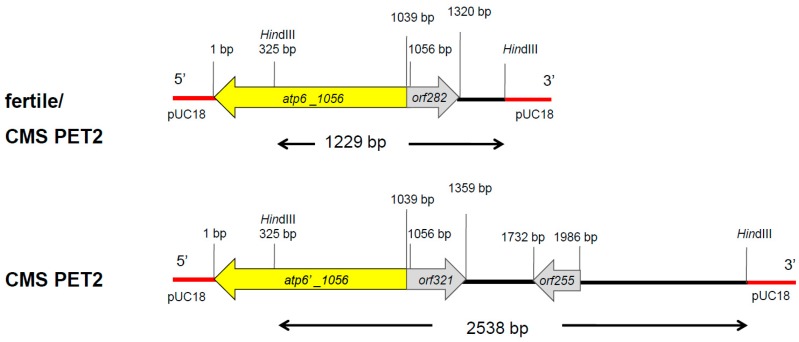
Comparison of the organization of the open reading frames on the hybridization fragments obtained by using *atp6* as probe in the normal, fertile HA89 and CMS PET2.

**Figure 4:**
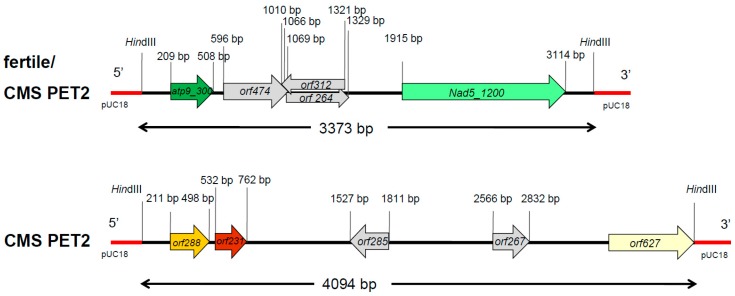
Comparison of the organization of the open reading frames on the hybridization fragments obtained by using *atp9* as probe in the normal, fertile HA89 and CMS PET2.

**Figure 5:**
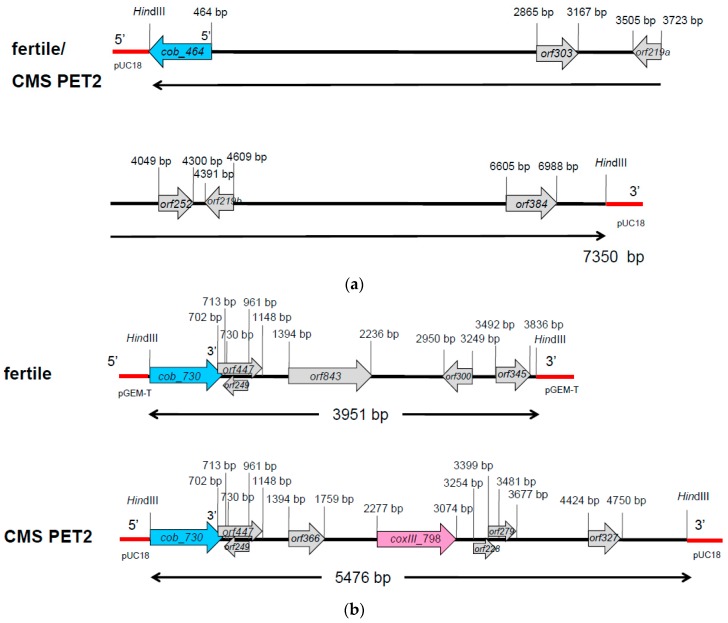
Comparison of the organization of the open reading frames on the hybridization fragments obtained by using *cob* as probe in the normal, fertile HA89 and CMS PET2. (**a**) 5′ cob fragment (7.3 kb) of fertile cytoplasm and CMS PET2, (**b**) 3′ cob fragment for the fertile cytoplasm (3.9 kb) and CMS PET2 (5.5 kb).

In Southern hybridizations using *Hind*III as restriction enzyme, CMS PET2 showed identical fragment patterns with CMS PET1 and the male fertile line HA89 for *atp8*, *coxIII* and *nd5* as probes. For the mitochondrial genes *atp6*, *atp9* and *cob,* an additional fragment was present in CMS PET2, apart from the fragments detected for HA89 and CMS PET1 (Figure 2). To establish more precisely the alterations present in CMS PET2, all fragments were cloned and sequenced. In total, 35 open reading frames (>201 bp) were detected, six corresponding to the mitochondrial genes used as probes and 12 were only present in the CMS PET2-specific fragments (Table S1). The organization of all open reading frames is shown in detail in Figure 3, Figure 4 and Figure 5. For *atp6* as probe, CMS PET2 showed a fragment of 1.2 kb identical to CMS PET1 and the male-fertile line HA89. The additional CMS PET2-specific fragment of 2.5 kb carried a second intact copy of *atp6′* (1056 bp) and two different orfs, *orf321* and *orf255* (Figure 3). For *atp9* as probe, one identical fragment of 3.4-kb was present in CMS PET1, fertile and CMS PET2, and an additional fragment of 4.1-kb was only visible in CMS PET2. The 4.1-kb-fragment contained a split *atp9* gene, which resulted in two new open reading frames of 228 bp and 231 bp, and three additional orfs, *orf285*, *orf267* and *orf627* (Figure 4). Hybridization with *cob* as probe showed the same fragment of 7.3 kb in all cytoplasms carrying the 5′ end of *cob* (Figure 5A), as well as a 3.9-kb-fragment for the fertile cytoplasm and CMS PET1, but a 5.5-kb-fragment for CMS PET2 carrying the 3′ end of *cob* (Figure 5B). A recombination within *orf843* resulted in a shortened *orf366* and an enlarged *Hind*III fragment in CMS PET2. Seven additional orfs could be identified on this fragment, one of these represents *coxIII*.

Comparison of the fragment sequences with the mitochondrial sequence of HA412 (accession no KF815390) revealed that the fragments present in the male-fertile, normal HA89 and in CMS PET2 were 99–100% homologous to HA412 (Table S2). For the CMS PET2 specific 2.5-kb-fragment (*atp6*) a recombination between two identical areas (326 bp in size) in the mitochondrial DNA seems to be the reason for the larger fragment.

Also, the 5.5-kb-fragment specific for CMS PET2 (*cob*) represents a recombination event between two mtDNA regions, even though there is a small area with no homology between them. However, the 4.1-kb-fragment specific for CMS PET2 (*atp9*) represents a scramble of four small fragments with homology to mitochondrial sequences (>100 bp and <600 bp in size) interrupted by sequences with no homology to the mtDNA.

To answer the question if the detected 12 open reading frames in the additional CMS PET2-specific fragments (Table S1) were unique for CMS PET2 or moved via recombination from other places to these new locations, all orfs present on the CMS PET2-specific fragments were also amplified by PCR with sequence tagged site primers (Table S3) in *H. annuus*, *H. petiolaris*, CMS PET2 and the fertility-restored hybrid CMS PET2 × IH-51. Five open reading frames—*orf288*, *orf231*, *orf285*, *orf267* and *orf627—*proved to be unique to CMS PET2 (Figure S1). All of these were localized in the 4.1-kb-fragment of CMS PET2, which had hybridized to *atp9*. These orfs were also present in the fertility-restored hybrid.

### 2.2. Origin of Orf288 and Orf231 in CMS PET2

**Figure 6:**
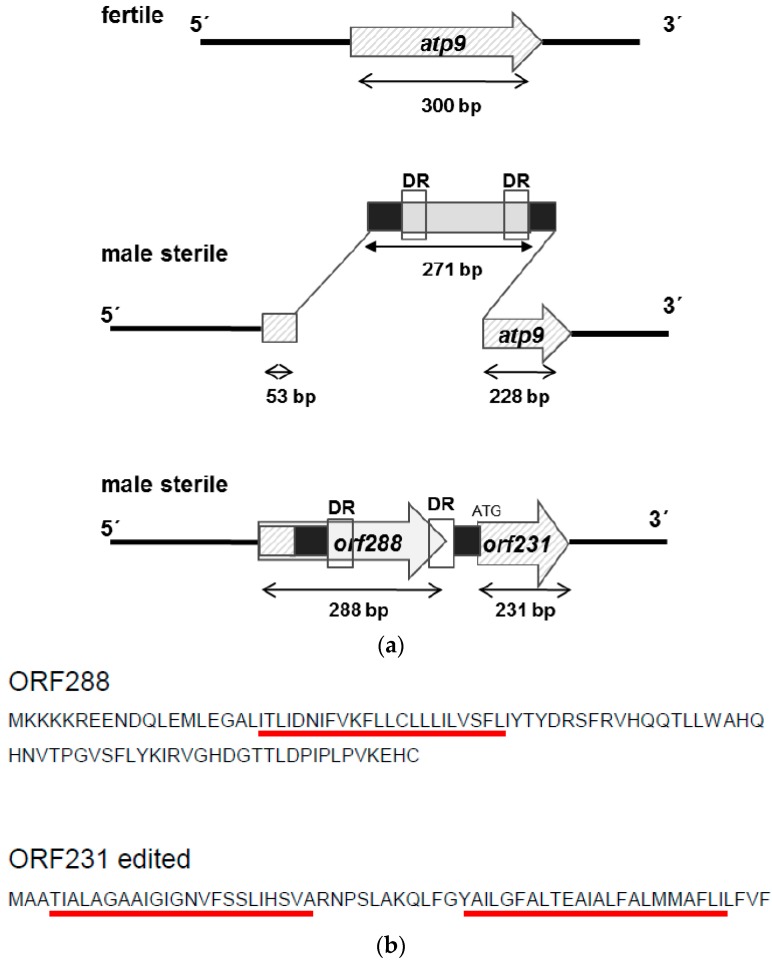
Model for the creation of the CMS-PET2 specific *orf288* and *orf231* by an insertion event of 271 bp into the duplicated *atp9* gene. (**a**) Scheme; (**b**) Amino acid sequences of *orf288* and *orf231* (edited). Transmembrane domains as predicted by TMHMM are marked by red bars.

After the duplication of the *atp9* gene, an insertion of 271 bp occurred in the 5′ coding region of the *atp9* gene (Figure 6A), creating two new open reading frames, *orf288*, coding for a potential protein of 11.1 kDa, and *orf231*, encoding a 7.9-kDa-protein. Blast analyses indicated that the insertion represents a unique sequence not present elsewhere in genomes. *Orf231* showed 87.4% homology to the *atp9* gene of sunflower. Comparison between the *orf288* and *orfH522*, responsible for male-sterile phenotype in CMS PET1, showed 33.3% homology and *orf288* versus *orfB* 35.3%. These results underline the specificity of *orf288* to CMS PET2. It is interesting to note that the first 53 bp of *orf288* are identical with the 5′ coding region of the *atp9* gene in the CMS PET1 cytoplasm and the male-fertile line HA89. Moreover 19 bp of *atp9* were deleted by the insertion event and the last three base pairs of the 271-bp-insertion act as start codon for *orf231*, which otherwise consists of the 5′ deleted *atp9* (−72 bp/+3 bp) located 33 bp downstream of *orf288*. Furthermore, a small direct repeat of 10 bp (ACTGCTAATC) could be found inside of the 271-bp-insertion (Figure S2). To characterize the protein encoded by *orf288*: it encodes a protein of 95 aa (pI 7.84, Mw 11.1 kDa), of which 16.9% are basic and 10.6% acidic amino acids including a transmembrane domain of 23 aa (Figure 6B).

### 2.3. Expression and RNA Editing of Orf288 and Orf231

**Figure 7:**
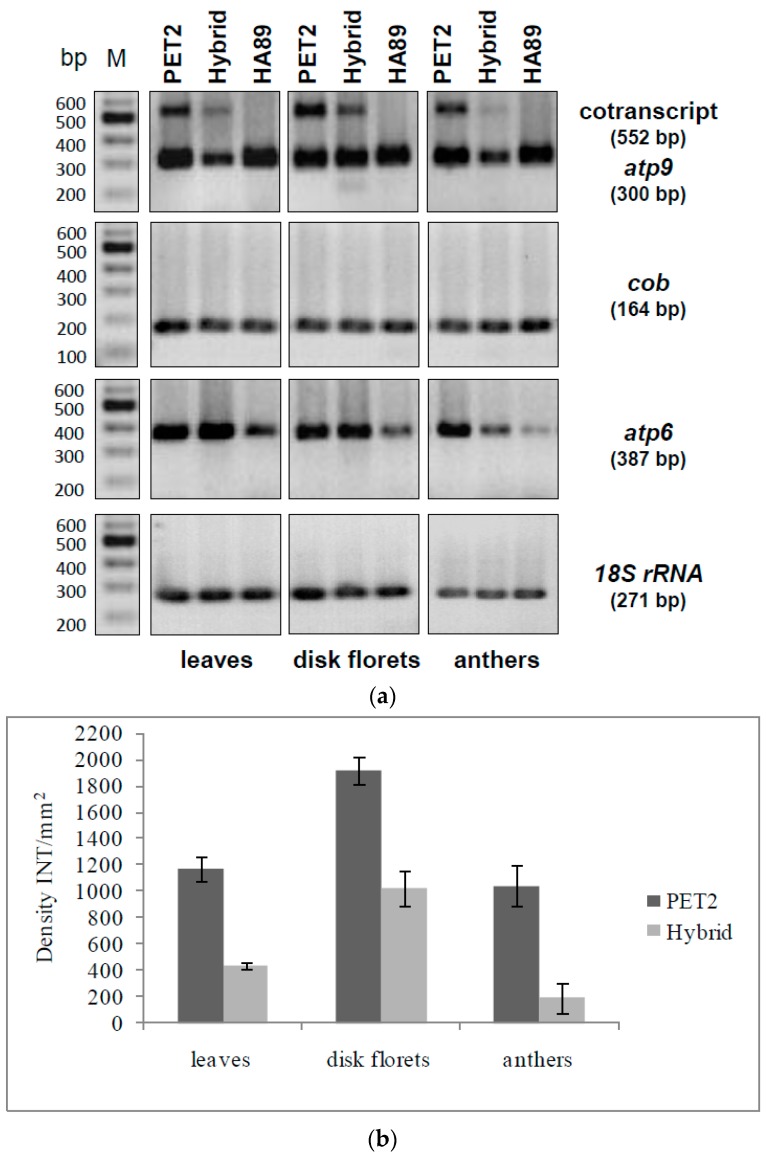
RT-PCR analysis of CMS PET2, the fertility-restored hybrid and the male-fertile line HA89 (**a**) PCR amplification products using primer specific for *atp9* (*orf300*), *cob* (*orf1194*), *atp6* (*orf1056*) and *18S rRNA*. Expected fragment sizes are given in brackets. Lanes: 1, CMS PET2 (male sterile); 2, PET2 × IH-51 (fertile F1-hybrid); 3, HA89 (fertile, normal cytoplasm); M, 100 bp marker; (**b**) Quantification of the co-transcript expression level (552 bp) in CMS PET2 and the fertility-restored hybrid in leaves, disk florets and anthers by densitometry. Dark grey: CMS PET2, light grey: fertility-restored hybrid (CMS PET2 × IH-51).

In order to characterize *orf288* and *orf231* as potential candidates for male sterility in CMS PET2, semi-quantitative RT-PCRs were performed. Mitochondrial *18S rRNA* was used as internal standard and *atp9* (*orf300*), *atp6* (*orf1056*) and *cob* (*orf1194*) as references. In leaves, disk florets and anthers unique signals for the co-transcript of *orf288* and *orf231* (552 bp) were only detected in CMS PET2 and the fertility-restored hybrid (Figure 7A). On the other hand, expression of *atp9* (*orf300*), *atp6* (*orf1056*) and *cob* (*orf1194*) occurred in CMS PET2, the fertility-restored hybrid as well as the male fertile line HA89 without any observable differences in signal intensity.

In sunflower, 11 editing sites had been described for the *atp9*-mRNA [44], and these were also found in the new *orf231* (Figure S3). All sites are fully edited as in *atp9*, resulting in a protein of 64 amino acids with a molecular weight of 6.7 kDa (pI 8.37, Figure 6B). Inspecting *orf288*, no editing sites could be identified. In contrast to the high signal intensity in CMS PET2, the co-transcript of *orf288* and *orf231* was significantly reduced in the fertility-restored hybrid and not present in the male-fertile line HA89 (Figure 7A). Quantification of the signal intensity revealed a strong down-regulation of the co-transcript (552 bp) in the fertility-restored hybrid by 2.7 in leaves, by 1.9 in disk florets and by 5.4 in anthers in comparison to CMS PET2 (Figure 7B).

### 2.4. Presence of Orf288 and Orf231 in CMS GIG1

Southern hybridization had grouped CMS PET2 and CMS GIG1 together into the MT-γ group [19]. Therefore it was interesting to see if CMS GIG1 also contained *orf288* and *orf231*. Cloning and sequencing of the two PCR products (743 and 491 bp) obtained by the primer combination PET2spec_for/PET2spec_rev identified these two open reading frames also in CMS GIG1 (Figure S4). The second functional copy of the *atp9* was also present. The sequences of *orf288*, *orf231* and *atp9* are 100% identical between CMS PET2 and CMS GIG1. This confirms the relevance of *orf288* and *orf231* for the male sterile phenotype in the presence of these two CMS cytoplasms.

### 2.5. Protein Analyses of Orf288 and Orf231

**Figure 8:**
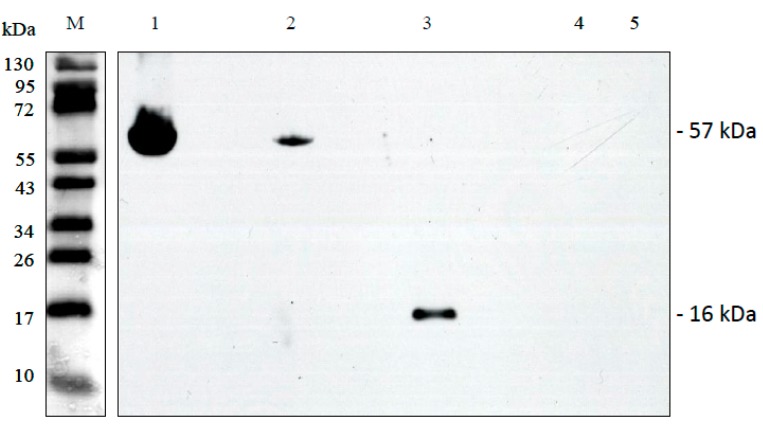
Immuno-blot using complete affinity-purified peptide Anti-ORF288 polyclonal antibody against 10 µg of isolated mitochondria from CMS PET2 and the fertile line HA89, ORF288 recombinant protein and protein plant extracts of CMS PET2 and HA89. Lanes: 1, CMS PET2 mitochondrial extracts; 2, HA89 mitochondrial extracts; 3, recombinant ORF288; 4, CMS PET2 whole plant protein extracts; 5, HA89 whole plant protein extracts; M, prestained protein ladder.

For biochemical verification, ORF288 and ORF231 were produced as recombinant proteins after overexpression in *E. coli* using the vector pET28a. Induction of the expression by IPTG reduced the growth of the bacteria heavily indicating a cytotoxic effect of both proteins (data not shown). In both cases, the His-tagged proteins were purified by affinity chromatography on Ni-NTA columns, but this was hampered by the fact that the two membrane proteins ORF288 and ORF231 were overexpressed in form of inclusion bodies. Immuno-blotting analysis using the specific Anti-ORF288 antibody, raised against a peptide derived from ORF288, revealed a strong signal of 57 kDa in CMS PET2 and a much weaker of the same size in HA89 (Figure 8). A specific band of 11 kDa, representing the ORF288 in isolated CMS PET2 mitochondria and protein plant extracts was not observed, but a signal of 16 kDa serving as positive control for antibody reaction against the recombinant protein. It is conceivable that the 57-kDa-signal represents an aggregation of six to eight subunits of ORF288 or that ORF288 was enclosed in another multimeric structure.

### 2.6. Development of Diagnostic Markers for CMS PET1 and CMS PET2 Cytoplasm

**Figure 9:**
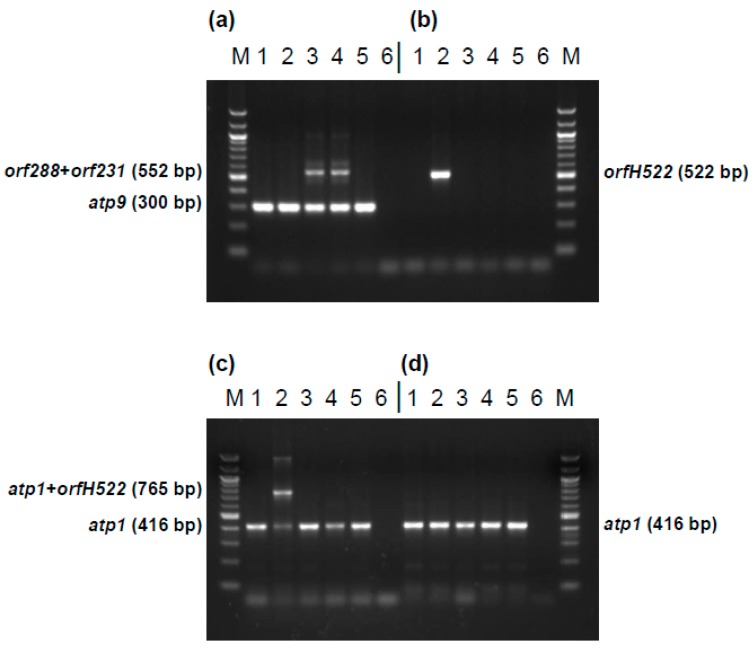
Development of markers specific for CMS PET1, CMS PET2 and the fertile, normal cytoplasm in sunflower. (**a**) Marker HRO_ATP9-PET2 using primer combination orf300_for/orf300_rev; (**b**) Marker HRO_PET1 using primers orfH522_for/orfH522_rev; (**c**) Marker HRO_ATP1-PET1 using primer combination M_atp1_for/M_atp1_rev/orfH522_rev; (**d**) Marker HRO_ATP1 using primer combination M_atp1_for/M_atp1_rev; Lanes: 1, HA342 (male fertile, normal cytoplasm); 2, CMS PET1 × HA342 (male sterile); 3, CMS PET2 × IH51 (fertility restored F1-hybrid); 4, CMS PET2 × RHA265 (male sterile); 5, RHA265 (restorer line, male fertile, normal cytoplasm); 6, PCR negative control; M, 100 bp marker.

To use different CMS cytoplasms in commercial sunflower hybrid breeding it is essential to distinguish the CMS sources by diagnostic markers. For this purpose four markers were developed (Figure 9): (1) marker HRO_ATP9-PET2 distinguishing the normal, fertile cytoplasm from CMS PET2, (2) marker HRO_PET1 specific for CMS PET1, also showing the absence of *orfH522* in CMS PET2 (3) marker HRO_ATP1-PET1 detecting CMS PET1 in combination with the internal *atp1* control, and (4) marker HRO_ATP1 as internal PCR control present in all cytoplasms. Application of these four diagnostic markers allows a clear differentiation between CMS PET1, CMS PET2 and the fertile cytoplasm in sunflower.

## 3. Discussion

### 3.1. Molecular Mechanisms behind Male Sterility in CMS PET2

In this study, a correlation between male sterility in CMS PET2 and mitochondrial rearrangements involving the *atp9* gene was identified. In addition, CMS PET2 showed a duplication of *atp6* and a recombination in the second *cob*-specific fragment. However, only the co-transcript of the split second copy of *atp9*, creating two new open reading frames *orf231* and *orf288*, showed a clear reduction of the co-transcript in the fertility-restored hybrids. This reduction of the co-transcript, especially in the anthers of fertility-restored hybrids, indicates an involvement in male sterility. This raised the question, how the proteins encoded by the edited *orf231* (6.7 kDa) or *orf288* (11.1 kDa) and/or their co-transcript could be responsible for male-sterility. In a number of CMS systems, one of the first visible signs of CMS is the premature degeneration of the tapetum layer in the anthers [6]. In the PET1-mediated male-sterility this happens after meiosis II [24]. Release of cytochrome C from the mitochondria in the male-sterile lines leads to a premature programmed cell death [25]. The fact that the transcription rate of the co-transcript of *orf288* and *orf231* is reduced by 5.4 in the anthers of fertility-restored hybrids in comparisons to the male sterile PET2, indicates that reduction of the co-transcript in anther-specific tissues may play an essential role in restoring microspore development. Although the expression of *atp9*, which is highly edited in sunflower [44], is not changed in CMS PET2, the expression of *orf288* and *orf231* might interfere with the correct function of the membrane bound F0-part of the F1F0-ATP synthase due to the partial homology to *atp9* and could thereby be responsible for male sterility in CMS PET2. Lack of ATP would hamper the production of functional pollen, a highly energy demanding process [45]. However, investigations of the respiratory activity and the use of the alternative pathway did not show any weaknesses for CMS PET2 and CMS GIG1 in mitochondria isolated from etiolated seedlings [46]. This indicates that changes in the mitochondrial respiration activity might only be visible in the generative tissue due to interaction with flower-specific factors.

Overexpression of both open reading frames *orf231* and *orf288* leads to a reduction in bacterial growth and indicates that these two proteins might be cytotoxic in *E. coli* as observed for other CMS-specific proteins [10,31]. The CMS-specific protein of 12.4 kDa observed in the in organello translation products [15] could correspond to the 11.1-kDa-protein encoded by *orf288*. The edited normal *atp9* in sunflower would have a size of 9.2 kDa. It would thereby be smaller than the detected CMS-specific protein, but would have overlapped in the gels with a potential protein of 6.7 kDa encoded by an edited *orf231* mRNA. The antibody produced against the *orf288* peptide showed a strong band of 57 kDa in CMS PET2 and a much weaker in HA89 mitochondrial extracts. The peptide represents the first 14 amino acids of ORF288 and is therefore not only identical to ORF288 but also to ATP9, which explains the signal in HA89. The ATP9 protein tends to form oligomeric structures that are not separated by SDS treatment [47,48] explaining the signal with a larger complex. The signal in CMS PET2 is very strong indicating a higher accumulation of protein complex than in the male-fertile HA89. This complex might represent aggregations of ATP9, ORF288 or ORF231 or combinations of the proteins. In the Owen male sterile cytoplasm in sugar beet, an ATP9 ring structure, not yet assembled into complex V, was also observed [49]. In addition, free ATP9 was accumulated in male-sterile line and reduced upon fertility-restoration. Combining the results of Blue native gel electrophoresis, in-gel activity assays and LC-MS-MS-MS it could be demonstrated for the Owen cytoplasm that preSATP6, the CMS-specific component [50], interacts with the assembly and the activity of F1F0-ATPase, probably via the ATP9 protein [49]. Interestingly, the restorer-of-fertility *Rf1(X)* in sugar beet represents an unusual restorer gene, which encodes a homolog of an OMA1 protein [51]. OMA1 in combination with OXA1 interacts with ATP9 and supports the correct assembly of complex V [52]. Assuming that *Rf1(X)* in sugar beet acts in the same way, fertility restoration leads, by an unknown mechanism, to a reduction in ATP9, but not in preSATP6 [49]. Even accumulation of normal ATP9 may play a role in the CMS mechanism by effects on the mitochondrial proteome [49].

There are still interesting questions for CMS PET2 to be answered to fully understand the CMS mechanism. It would be interesting to see by Blue native gel electrophoresis whether products of *orf288* and *orf231* associate with the F1F0-ATP synthase or other complexes of the respiratory chain and thereby might interfere with the ATP production, especially in the anthers. As proof of concept transgenic tobacco plants expressing either *orf288* or *orf231* under the control of an anther-specific promoter and targeted to the mitochondria should be produced to see if the products of these orfs induce male sterility. 

### 3.2. Recombination Events Leading to Rearrangements at Atp9

Rapid changes in the genome structure via illegitimate recombination activity and emergence of novel split and chimeric gene structures, represent an apparent vulnerability of plant mitochondrial DNA and lead to CMS phenotypes in higher plants [33,53]. A 10-bp-direct repeat was observed within the 271-bp-insertion in the *atp9* gene in CMS PET2. This might be responsible for illegitimate recombination activity [54] and thereby involved in the creation of the male-sterile phenotype in CMS PET2. The use of the flanking regions of mitochondrial respiratory/ATP synthesis-related genes is typical for CMS-specific new orfs [4,6,54]. Especially, parts of *atp6*, *atp8* and *atp9* are most frequently involved in creating CMS-specific new open reading frames [1]. This seems to be also true for CMS PET2, where the insertion event of 271 bp uses the promoter and the first 53 bp of the 5′ coding region of the *atp9* gene to create *orf288*. In addition, the insertion provides an ATG start codon, which allows the creation of *orf231* by using the remaining 3′ part of the *atp9* and the termination signal. The *orf231* represents the C-terminal transmembrane part of *atp9* as does *orf77* associated with S cytoplasmic male sterility in maize [55]. CMS GIG1, which grouped together with CMS PET2 in the MT-γ group [19], showed the same hybridization pattern as CMS PET2 with *atp9*, *atp6* and *cob* as probes. This was surprising as well as the presence of *orf288* and *orf231* in CMS GIG1 because of the different origin of the CMS sources. CMS GIG1 resulted from an interspecific cross of *H. giganteus* and *H. annuus* [23]). However, also the PET1-like cytoplasms have different origins but the same CMS-mechanism [22]. In addition, for CMS PEF1, which originates from an interspecific cross of *H. petiolaris* ssp. *fallax* with *H. annuus* [56], a modification at the *atp9* gene seems to be responsible for male sterility [13]. Here a 500-bp-insertion in the 3′ UTR of the *atp9* gene was identified as cause. Alterations of the *atp9* gene region were also found in *Daucus carota* [57,58], in *Brassica napus* ‘Tournefortii-Stiewe’ [59] as well as *Boehmeria nivea* [60] and may result in dysfunctions of mitochondria in form of insufficient supply of ATP for pollen development.

### 3.3. Role of RNA Editing in Creating a Functional ATP9

RNA editing, a posttranscriptional process, which is essential to produce functional proteins as it frequently leads to amino acid exchanges, was observed for *atp9*. In this process the genetic information is typically changed from C-to-U on mRNA level by deamination [61,62,63]. The *atp9*-mRNA represents one of the best studied RNA editing objects. In most plants like *Oenothera* [64] and potato [65], it is highly edited. Changes in RNA editing can be involved in creating CMS because of amino acid transitions like S to L, P to L and S to F as consequence of the process [65]. In the case of *atp9*, RNA editing is required for creating a functional protein [66]. The fatal role of abnormal *atp9* transcripts for pollen development has been demonstrated in transgenic tobacco and *Arabidopsis thaliana* plants by expressing the unedited *atp9* gene and targeting it to mitochondria [67,68]. Mitochondrial dysfunctions affected normal anther development, especially the fate of the tapetum cell layer and reduced pollen formation [69]. RNA editing tends to increase the proportion of hydrophobic amino acids like leucine, which are crucial for complex formation as well as integrity of proteins [70]. In mitochondria unedited ATP9 protein involved in the proton channel may affect the functioning of F1F0-ATP synthase and thereby reduce the ATP production. However, the CMS PET2-specific *orf231*, which carries all 11 editing sites of *atp9* in sunflower [44], is fully edited.

### 3.4. CMS PET2 as Alternative to CMS PET1 in Commercial Hybrid Breeding

An alternative CMS source for commercial sunflower hybrid breeding would require that it is based on a different CMS mechanism than CMS PET1. The fact that RHA265, a restorer line of CMS PET1, represents a maintainer line of CMS PET2 [14], has already been a good indicator that CMS PET2 is different from CMS PET1 with regard to the CMS mechanism. In addition, clear differences in the anther morphology between plants carrying CMS PET2 or CMS PET1 also pointed to another mechanism leading to male sterility in CMS PET2. Previous studies on CMS PET1 had shown that a 17-kb-region of the mitochondrial genome, a 12-kb-inversion and a 5-kb-insertion/deletion, flanked by 261-bp inverted-repeats, are involved in PET1 male-sterility [27,71]. The 5-kb-insertion created the new *orfH522* downstream of *atp1* that encodes a 16-kDa-protein, which accumulates in male-sterile and fertility-restored CMS PET1 seedlings [26,28]. The results obtained so far for CMS PET2 indicate that the molecular mechanisms behind the PET2 male-sterility in sunflower depends on recombination events involving the *atp9* gene, which lead to the two new open reading frames *orf288* and *orf231*. In addition, no PCR signal for *orfH522* was visible in CMS PET2. Thereby CMS PET2 clearly differs from the mechanism in CMS PET1.

To use CMS PET2 as alternative male sterility sources in commercial hybrid breeding requires restorer lines with very good fertility restoration capacity and markers to introduce the fertility restorer gene into a breeding pool. In test crosses using five restorer lines of CMS PET1, the line IH-51 produced 100% fertile plants when CMS PET2 was used as mother [14]. Segregation analyses showed that a single restorer gene is responsible for fertility restoration. AFLP markers linked to the restorer gene *Rf_PET2*, which is also located on linkage group 13 as *Rf1*, were identified [72].

Easy differentiation of cytoplasms is essential if different CMS sources are intended to be used in hybrid breeding. This study here, also presents molecular markers distinguishing CMS PET1, CMS PET2 and the fertile normal cytoplasm in sunflower by easy to use simple PCR-markers. Also in *Raphanus sativa* markers were developed to differentiate the Ogura cytoplasm from other mitochondrial types and to identify novel sub-stoichiometric organizations [73]. In addition, 12 CMS-specific markers as well as SSR-markers were developed to discriminate the mitochondrial genomes present in six *Brassica* species [74]. Also, in *Gossypium hirsutum*, Zhang et al. [75] obtained SCAR and SSR markers to differentiate between CMS and maintainer lines.

Improvements in important agronomical traits can get neglected by restricting hybrid breeding to a single CMS cytoplasm. In potato, cytoplasm specific markers were used to characterize 1217 European potato cultivars with regard to the presence of the six known cytoplasm types (T, D, W, A, M and P) [76]. With regard to agronomic important traits, the W-cytoplasm could be correlated with increased tuber starch content and later maturity whereas the D- and M-type of cytoplasm showed more resistance towards late blight [76]. Diversification on the cytoplasm side can help to improve the agronomic performance of a crop and to reduce the vulnerability to pathogens. The results presented in this study may be the first steps to use more than the CMS PET1 cytoplasm in sunflower commercial hybrid breeding.

## 4. Materials and Methods

### 4.1. Plant Material

Three male-fertile sunflower inbred lines, HA89 (maintainer line of CMS PET1 and CMS PET2), RHA265 (maintainer line of CMS PET2, restorer line of CMS PET1), and IH-51 (restorer line of CMS PET2), three CMS-lines PET1 [8], PET2 [21], GIG1 [23], and the fertility-restored hybrid PET2 (RHA265) × IH-51 were used in this study. The CMS lines were obtained from Hervé Serieys within the FAO program [12]. The plants were cultivated in the greenhouse under controlled conditions.

### 4.2. DNA Isolation and Labeling

Total genomic DNA was extracted from leaves according to the protocol of Doyle and Doyle [77]. Mitochondrial DNA was isolated using the procedure of Horn [78]. The mitochondrial genes *atp6* (subunit 6 of the ATPase gene of sunflower), *atp9* (subunit 9 of the ATPase gene of sunflower) and *cob* (apocytochrome b gene of sunflower) were used as probes after amplification with gene specific primers (Table S3). Primers were designed using web primer (available online: http://www.yeastgenome.org/cgi-bin/web-primer). The probes were labeled, using three different labeling systems: ECL Direct™ Nucleic Acid Labeling and Detection System (Amersham, GE Healthcare, Munich, Germany), ^32^P radioactively labeled overgo-primer (Hartmann Analytic GmbH, Braunschweig, Germany) and Prime-It II Random Primer Labeling Kit (Agilent Technologies, Santa Clara, CA, USA) according to the supplier’s instructions.

### 4.3. Cloning and Sequencing

The mitochondrial DNA was digested with *Hind*III restriction endonuclease (Fermentas, St. Leon-Rot, Germany), separated on a 0.8% agarose gel and blotted on Hybond N+ membrane (Amersham, GE Healthcare, Munich, Germany) using the procedure of Evans et al. [79] (1994). Hybridization with *atp6*, *atp9* and *cob* as probes using ECL Direct™ Nucleic Acid Labeling and Detection System (Amersham, GE Healthcare, Munich, Germany) according to the manufacturer’s recommendations followed. Blots were washed in 0.5 × SSC, 0.4% SDS, 6 M urea at 42 °C for 20 min two times, 2 × SSC, 42 °C for 5 min and exposed to Amersham Hyperfilm ECL (Amersham, GE Healthcare, Munich, Germany) for 0.5–6 h. The *Hind*III digested mtDNA was cloned into pUC18 vectors and the resulting recombinant plasmids were used to prepare a mitochondrial DNA library. Positive clones were selected by *Hind*III hybridization pattern with *atp9*, *atp6* and *cob* as probes (Table S3) and sequenced. The 3.9-kb-*cob* fragment from HA89 was amplified by PCR and cloned into the pGEM-T Easy vector according to the manufacturer’s protocol (Promega, Mannheim, Germany). Blast searches against the NCBI database (available online: http://blast.ncbi.nlm.nih.gov/Blast.cgi) and ORF-Finder program (available online: https://www.ncbi.nlm.nih.gov/orffinder/) were used to detect homologies and open reading frames. Molecular weights and isoelectric points were calculated online (available online: http://web.expasy.org/compute_pi/). Transmembrane domains were predicted using the TMHMM Server v.2.0 (available online: http://www.cbs.dtu.dk/services/TMHMM-2.0/).

### 4.4. RNA Isolation and Reverse Transcriptase (RT)-PCR Analysis

Total RNA was extracted from leaves using TRI Reagent (Sigma-Aldrich Biochemie GmbH, Hamburg, Germany). The RNA extraction from anthers and disk florets was performed by using RNeasy Plant Mini Kit (Qiagen, Hilden, Germany) according to the manufacture’s manual. The reverse transcription reaction (RT) was realized with 1 µg RNA and 200 U RevertAid™ H Minus M-MuLV reverse transcriptase (Fermentas, St. Leon-Rot, Germany), routinely carried out at 42 °C followed by incubation at 70 °C for 10 min. Each PCR-experiment was accompanied by the following controls, a reaction in which: (1) no RNA or DNA template was added, (2) DNase treated RNA was added and (3) RNA but no reverse transcriptase was added. All reactions did not yield detectable product. Semiquantitative RT-PCR were performed using the primers given in Table S3. RNA loading was standardized relative to sunflower mitochondrial 18S rRNA.

### 4.5. Overexpression of Recombinant Orfs in E. coli

The *orf288* and *orf231* (5′ deleted *atp9*) were amplified by PCR using the gene-specific primers with corresponding cleavage sites for *Sac*I/*Hind*III (Table S3). PCR fragments were cloned into pGEM-T Easy vector according to the manufacturer’s protocol (Promega, Mannheim, Germany) and sequenced with T7 primer. DNA from verified plasmids was cut with *Sac*I/*Hind*III and the fragments were cloned into the expression vector pET28a (Novagen, Madison, WI, USA). *E. coli* BL21 strains expressing a fusion protein were grown in LB medium to an OD600 of 0.6. The gene expression was induced by addition of 1 mM IPTG. The fusion proteins carried N-terminal His-tags for purification with the Ni-NTA Fast Start Kit, according to the protocol of Qiagen (Hilden, Germany). The cells were harvested and resuspended in 10 mL digestion buffer pH 8.0 containing 50 mM NaH_2_PO_4_, 300 mM NaCl and 1mg/mL lysozyme. The protein was extracted by ultrasonic treatments (4 × 30 s, 90 W) under ice-cooling. Afterwards efficient purification from cleared *E. coli* lysates was done with Ni-NTA columns under denaturing conditions. The eluted proteins were checked regarding purity using SDS-PAGE.

### 4.6. Immuno-Blotting

A complete affinity-purified peptide Anti-ORF288 polyclonal antibody was produced by GenScript USA (Piscataway, NJ, USA) against the peptide MKKKREENDQLEMC, representing the first 14 amino acids of *orf288* plus a C added for KLH conjugation. For the Western blot, 10 µg of recombinant protein and mitochondrial protein extracts from CMS PET2 and the fertile line were separated in 12% Tris-Tricine gels and blotted onto nitrocellulose membranes (Amersham, GE Healthcare, Munich, Germany). After visualization by Ponceau S staining (0.2% Ponceau S in 0.25% acetic acid) the membrane was blocked in TBS-Triton pH 7.6 (2.42 g Tris, 8 g NaCl, 2.5 mL 20% Triton) with 5% skim milk for 2 h at 25 °C and incubated with the antisera after 10fold dilution in TBS-Triton with 5% skim milk overnight at 4 °C under shaking. After washing with TBS-Triton the secondary peroxidase-labeled anti-rabbit IgG HRP-linked antibody (GE Healthcare, Munich, Germany) diluted 1:2000 was added for 2 h at 25 °C under shaking. The blots were exposed to Amersham Hyperfilm ECL (Amersham, GE Healthcare, Munich, Germany).

### 4.7. Accession Numbers

The online available accession numbers (https://www.ncbi.nlm.nih.gov/) for *Helianthus annuus* are: *atp6* (X82388), *atp9* (X51895), *nd5* (AF258785.1), *orfB* (X57669.1), *coxIII* (X57669), *cob* (X98362), *orfH522* (X55963), *18S rRNA* (AF107577), complete mitochondrial genome cultivar HA412 (KF815390.1). Sequences of the *Hind*III fragments obtained with *atp6*, atp9*atp9* and *cob* as probe for CMS PET2 and HA89 were deposited in Genbank under the accession numbers MF828616-MF828625.

## Supplementary Materials

Supplementary materials can be found at http://www.mdpi.com/1422-0067/19/3/806/s1.
