# Supplementary material for: Recombination Events Involving the atp9 Gene Are Associated with Male Sterility of CMS PET2 in Sunflower
Source: Int J Mol Sci. 2018 Mar 11;19(3):806. doi: 10.3390/ijms19030806 (PMC5877667; doi:10.3390/ijms19030806)
Supplement: Supplementary file 1 [file ijms-19-00806-s001.doc]

**Table S1:** Complete list of the detected open reading frames (> 201 nt) using Blastx searches against the NCBI database and ORF-Finder program. Fertile = HA89, sterile= CMS PET2

| Probe |  | ORF [bp] | Start – Stop [bp] | kDa | Frame | Homology to | Organism | Blastx e-value | Accession |
| --- | --- | --- | --- | --- | --- | --- | --- | --- | --- |
| ***atp6*** | fertile  1.2 kb | 1056 | 1056-1 | 38.6 | -2 | *atp6* | *H. annuus* | 8e-133 | ACR54092 |
|  |  | 282 | 1039-1320 | 10.2 | 1 | - |  | 2.0 | - |
|  | sterile  2.5 kb | 1056 | 1056-1 | 38.6 | -2 | *atp6* | *H. annuus* | 8e-133 | ACR54092 |
|  |  | 321 | 1039-1359 | 11.7 | 3 | - |  | - | - |
|  |  | 255 | 1986-1732 | 9.4 | -2 | - |  | - | - |
| ***atp9*** | fertile  3.4 kb | 1200 | 1915-3114 | 44 | 1 | *nd5* | *H. annuus* | 0.0 | AAG23623 |
|  |  | 474 | 596-1069 | 17.3 | 2 | unnamed protein product | *B. vulgaris subsp. vulgaris* | 3e-29 | CAA39307 |
|  |  | 312 | 1321-1010 | 11.3 | -1 | *orf104* | *V. vinifera* | 3e-45 | YP_002608359 |
|  |  | 300 | 209-508 | 10.9 | 2 | *atp9* | *H. annuus* | 1e-26 | AAG23624 |
|  |  | 264 | 1066-1329 | 9.7 | 1 | hypothetical protein | *R. communis* | 3e-44 | YP_002608359 |
|  | sterile  4.1 kb | 288 | 211-498 | 11.1 | 1 | - | - | - | - |
|  |  | 285 | 1811-1527 | 10.5 | -3 | - | - | - | - |
|  |  | 267 | 2566-2832 | 9.7 | 1 | - | - | - | - |
|  |  | 231 | 532-762 | 7.9 | 1 | *atp9* | *H. annuus* | 7e-25 | AAG23624 |
|  |  | 627 | 3467>4093 | >24.6 | 2 | *DNA polymerase type B, organellar* | *Theobroma cacao* | 2e-126 | XP_017985381.1 |
| ***cob*** | fertile/  sterile  7.3 kb | 1194* | 467>1 | 43.8 | -3 | *5’ end cob* | *H. annuus H. petiolaris* | 0.0 | CAA67007.1 CAA84545.1 |
|  |  | 384 | 6605-6988 | 14 | 2 | *-* | - | - | - |
|  |  | 303 | 2865-3167 | 11 | 1 | - | - | - | - |
|  |  | 252 | 4049-4300 | 9.1 | 2 | - | - | - | - |
|  |  | 219a | 3723-3505 |  | -1 |  |  |  |  |
|  |  | 219b | 4609-4391 |  | -3 |  |  |  |  |
|  | fertile  3.9 kb | 1194* | <1-730 | 43.8 | 2 | *3’end cob* | *H. annuus H. petiolaris* | 0.0 | CAA67007.1 CAA84545.1 |
|  |  | *843* | 1394-2236 | 31.8 | 2 | *atp8* | *H. annuus* | 6e-82 | YP_008999555.1 |
|  |  | *447* | 702-1148 | 16.4 | 3 | *orf124* | *B. vulgaris subsp. vulgaris* | 1e-11 | NP_064106.1 |
|  |  | *345* | 3492-3836 | 13.2 | 3 | - | - | - | - |
|  |  | *300* | 3249-2950 | 11.1 | -1 | - | - | - | - |
|  |  | *249* | 961-713 | 8.7 | -3 | - | - | - | - |
|  | sterile  5.5 kb | 1194* | <1-730 | 43.8 | 2 | *3‘ end cob* | *H. annuus H. petiolaris* | 0.0 | CAA67007.1 CAA84545.1 |
|  |  | 798 | 2277-3074 | 29 | 3 | *coxIII* | *H. annuus* | 2e-94 | X57669 |
|  |  | 447 | 702-1148 | 16.4 | 3 | *orf124* | *B. vulgaris subsp. vulgaris* | 1e-11 | NP_064106.1 |
|  |  | 366 | 1394-1759 | 13.4 | 2 | *atp8* | *H. annuus* | 4e-47 | X57669 |
|  |  | 327 | 4424-4750 | 11.9 | 2 | - | - | - | - |
|  |  | 279 | 3399-3677 | 10.1 | 3 | hypothetical  protein | *Cucumis sativus* | 2e-42 | KGN44549.1 |
|  |  | 249 | 961-713 | 8.7 | -1 | - | - | - | - |
|  |  | 228 | 4623-4441 | 8.8 | -2 | succinate-oxido-reductase subunit 4 | *Platanus occidentalis* | 4e-30 | AAW47281.1 |

*size is given for the whole orf represented by two parts

**Table S2:** Overview of the homology in the *Hind*III fragments (> 100 bp; < e-10; >80% identity) to mitochondrial DNA sequence of HA412 (accession No. KF815390.1), bold – regions that represent the whole fragment in HA89 and CMS PET2; italics – region most likely involved in the recombination creating the CMS-specific fragment

| Fragment | Line | Region | KF815390.1 | Identity | Gaps | e-Value |
| --- | --- | --- | --- | --- | --- | --- |
| **1.2 kb *atp6*** | **HA89 fertile/ CMS PET2** | **1-1229**  510-836  617-740  633-805 | **269655-268427**  35681-35354  106241-106119  260187-260034 | **1227/1229 (99%)**  322/328 (98%)  119/124 (96%)  140/173 (81%) | **0/1229 (0%)**  1/328 (0%)  0/124 (0%)  19/173 (10%) | **0.0**  2e-161  8e-53  4e-44 |
| 2.5 kb *atp6* | CMS PET2 | *1-836*  *510-2538*  617-740  633-805 | *269655-268820*  *35681-33654*  106242-106119  260187-260034 | *834/836 (99%)*  *2018/2030 (99%)*  119/124 (96%)  140/173 (81%) | *0/836 (0%)*  *3/2030 (0%)*  0/124 (0%)  19/173 (10%) | *0.0*  *0.0*  2e-52  8e-44 |
| **3.4 kb *atp9*** | **HA89 fertile/ CMS PET2** | **1-111476** | **114848-111476** | **3373/3373 (100%)** | **0/3373 (0%)** | **0.0** |
| 4.1 kb *atp9* | CMS PET2 | *1-270*  *535-924*  536-726  *1204-1424*  *1884-2472* | *114848-114578*  *114568-114179*  98425-98239  *72816-73022*  *33772-34358* | *267/273 (98%)*  *387/390 (99%)*  153/191 (80%)  *187/221 (85%)*  *575/589 (98%)* | *5/273 (1%)*  *0/390 (0%)*  0/191 (0%)  *14/221 (6%)*  *2/589 (0%)* | *2e-129*  *0.0*  4e-44  *3e-64*  *0.0* |
| **7.4 kb *cob*** | **HA89 fertile/ CMS PET2** | **1-7351**  775-879 | **188913-181563**  113892-113996 | **7350/7351 (99%)**  104/105 (99%) | **1/7351 (0%)**  0/105 (0%) | **0.0**  4e-47 |
| **3.9 kb *cob*** | **HA89 fertile** | **1-3951**  1016-1745  1159-1423  2235-2422  3103-3241 | **188914-192864**  36393-37121  202900-202636  260766-260950  15064-15189 | **3951/3951 (100%)**  728/730 (99%)  263/265 (99%)  181/188 (96%)  114/140 (81%) | **0/3951 (0%)**  1/730 (0%)  0/265 (0%)  3/188 (1%)  15/140 (10%) | **0.0**  0.0  3e-133  1e-82  4e-31 |
| 5.5 kb *cob* | CMS PET2 | *1-1741*  *1859-5476*  1016-1798  1159-1423 | *188914-190658*  *37402-41014*  36393-37183  202900-202636 | *1739/1745 (99%)*  *3609/3618 (99%)*  780/791 (99%)  263/265 (99%) | *4/1745 (0%)*  *5/3618 (0%)*  8/791 (1%)  0/265 (0%) | *0.0*  *0.0*  0.0  5e-133 |

**Table S3:** Overview of the primers used for probe generation, cloning, RT-PCR and screening

| Primer | Gene/orf | Sequence 5’->3’ | Purpose |
| --- | --- | --- | --- |
| *atp6_for* | *atp6* | AAA GGA GGA GGA AAC TTA GTA CC | probe |
| *atp6_rev* |  | TCA TTC ATA CAT AGC ATA GTC CA |
| *atp9_for* | *atp9* | GGT GCA AAA TCA ATA GGG GCC G | probe |
| *atp9_rev* |  | ACC GAA TGA ATG CGT CAC AAG G |
| *cob_for* | *cob* | **AAG GAA CCA ACG ATT CTC GAT TC** | probe |
| *cob_rev* |  | **ATA TTG ATG CAA TGC GGC CAG** |
| *cob_for_F1* | *cob* 3.9 kb | GCTTTTATAGGATACGTACCACCTTG | cloning |
| *cob_rev_F3* |  | TGCTCATTCTAATGCTGGGAAG |
| *cob_for_F4* | *cob* 3.9 kb | TAGAGACTGGGCACCAAGAAAG | cloning |
| *cob_rev_F4* |  | TCAGCAAAGGAAAGAAGCCT |
| *orf231-fw-SacI* |  | GAG CTC ATG GCT GCT ACA ATT GC | Cloning |
| *orf231-rev-HindIII* |  | AAG CTT TTA TGA AAC TTC AAC CT |
| *orf288-fw-SacI* | *orf288* | GAG CTC ATG AAA AAG AAA AAG CG | Cloning |
| *orf288-rev-HindIII* |  | AAG CTT TTA GCA GTG CTC CTT TA |
| *orf231-for* | *orf231* | TAT TTA CTT TCG TTA TTT TTA TG | RT-PCR |
| *orf300-fw* | *atp9* | ATG AAA AAG AAA AAG CGT GAG GAG | RT-PCR |
| *orf300-rev* |  | TGC TTT ATG AAA CTT CAA CCT TC |
| *orf1056-fw* | *atp6* | GAG CTC ATG CCC AAC AAC TCC CAT G | RT-PCR |
| *orf1056-rev* |  | CCA TGG TTA ATG GAG ATT TAT AG |
| *orf1194-fw* | *cob* | GAG CTC GTG ACT ATA AGG AAC CAA | RT-PCR |
| *orf1194-rev* |  | CCA TGG CTA CTG GTA ATC ATT TG |
| *18S rRNA-fw* | *18S rRNA* | TGA TGG TAT CTT GCT ACT CGG ATA ACC | RT-PCR |
| *18S rRNA-rev* |  | CTC TCC GGA ATC GAA CCC TAA TTC TCC |
| *atp6_orf321_for* | *orf321* | ATGGGAGTTGTTGGGCATAA | Screening |
| *atp6_orf321_rev* |  | TTCCCGTATGTCATATCTCTTATTC |
| *atp6_orf255_for* | *orf255* | AGACTTGAATGAAGCAGCCTG | Screening |
| *atp6_orf255_rev* |  | TGAGCGAAGTAGGATCCTCTT |
| *atp9_orf288_for* | *orf288* | AGAAAAAGCGTGAGGAGAATG | Screening |
| *atp9_orf288_rev* |  | TTAGCAGTGCTCCTTTACCG |
| *atp9_orf231_for* | *orf231* | ATGGCTGCTACAATTGCTTCAG | Screening |
| *atp9_orf231_rev* |  | ATGAAACTTCAACCTTCTTTCTTTG |
| *atp9_orf285_for* | *orf285* | AAGGTGAGAAAAAGGACCGTC | Screening |
| *atp9_orf285_rev* |  | AGCATCCACTTGAGAATTTCC |
| *atp9_orf267_for* | *orf267* | TGAACCAATCTTTCGGTGAAGAG | Screening |
| *atp9_orf267_rev* |  | TGATAAATTGAGAGCAGTTCCTG |
| *atp9_orf627-for* | *orf672* | CAGGACATCCTCCTACTTGGT | Screening |
| *atp9_orf627_rev* |  | TTTCCAGCTTTCTTCGCTTC |
| *cob_orf366_for* | *orf366* | ATGTTGAAGGGGGCTGGTTG | Screening |
| *cob_orf366_rev* |  | CGCCTACTAAAAGAAAGGGGAG |
| *Cob_orf228_for* | *orf228* | CACCAAGAAATGACCCGAAA | Screening |
| *Cob_orf228_rev* |  | AAAGACAATTCCGAATCCGC |
| *Cob_orf279_for* | *orf279* | TTGCATTACTTATAGCTTCCTTG | Screening |
| *Cob_orf279_red* |  | AGTCATCAGTTCGAGCCTGATT |
| *Cob_orf327_for* | *orf327* | ATGGTCTGGGCTCTCACTCA | Screening |
| *Cob_orf327_rev* |  | TCAACTTGGATGGCTTGCTT |
| *cob_orf798_for* | *coxIII* | TCAGAGGCACTCTTATCATTTGG | Screening |
| *cob_orf798_rev* |  | CCCCACCAATAGATAGAGACAA |
| *orf300_for* | *atp9/PET2* | ATG AAA AAG AAA AAG CGT GAG GAG | 300 bp (fertile)/  + 552 bp (CMS PET2) |
| *orf300_rev* |  | TGC TTT ATG AAA CTT CAA CCT TC |
| *orfH522_for* | *orfH522* | TGCCTCAACTGGATAAATTCAC | 522 bp (CMS PET1) |
| *orfH522_rev* |  | ACCGTTCTCTCACGAGTTGAAG |
| *M_atp1_for* | *atp1* | TTATCTGTCAGTCGTGTTGGGTC | 416 bp (fertile)/  + 765 bp (CMS PET1) |
| *M_atp1_rev* |  | ATTGTGTAAGGCAAAGCGCA |
| *M_orfH522_rev* |  | TGCTTTGAGCATTTGGACCAG |


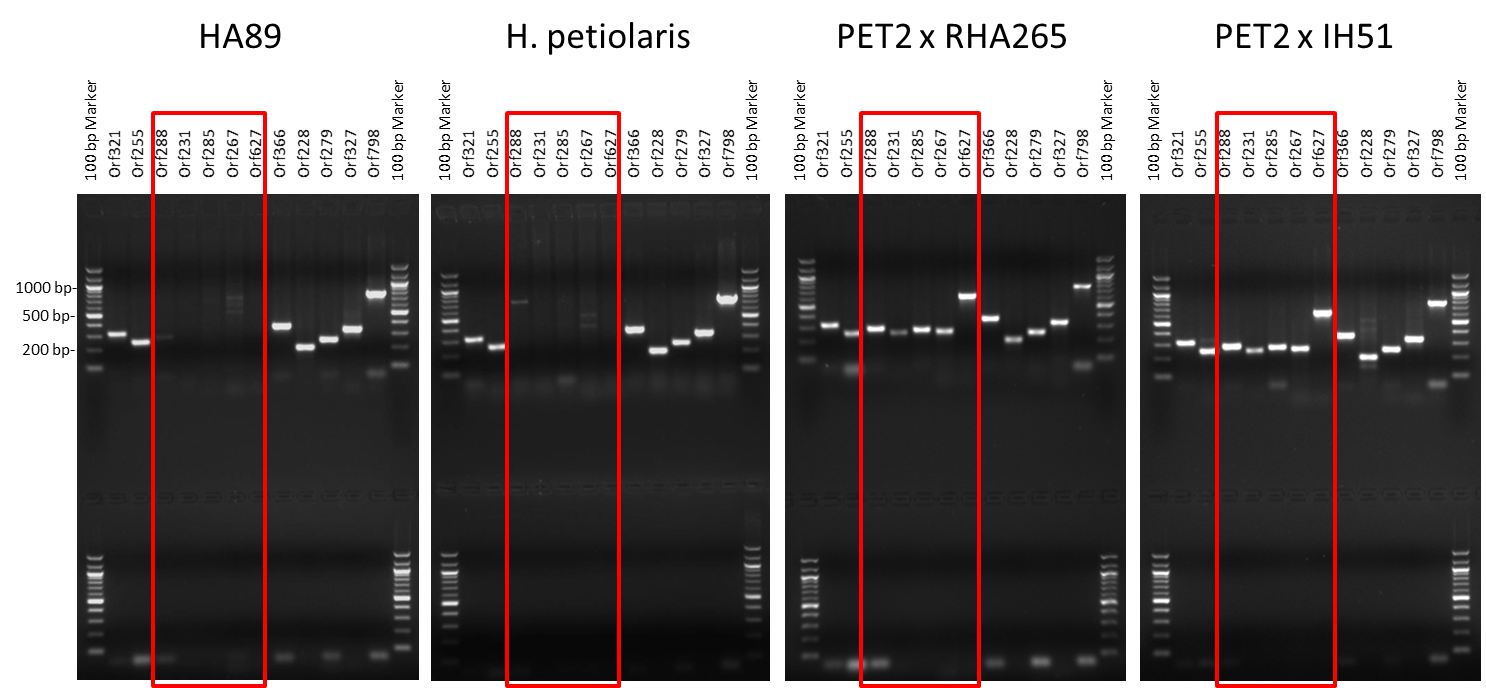


Figure S1: Screening for the presence of 12 open reading frames present in the CMS-PET2 specific fragments in HA89, *H. petiolaris*, CMS PET2 and the fertility-restored hybrid CMS PET2 x IH-51. Bottom part of the gels contains the negative controls for the primer combinations with H_2_O.


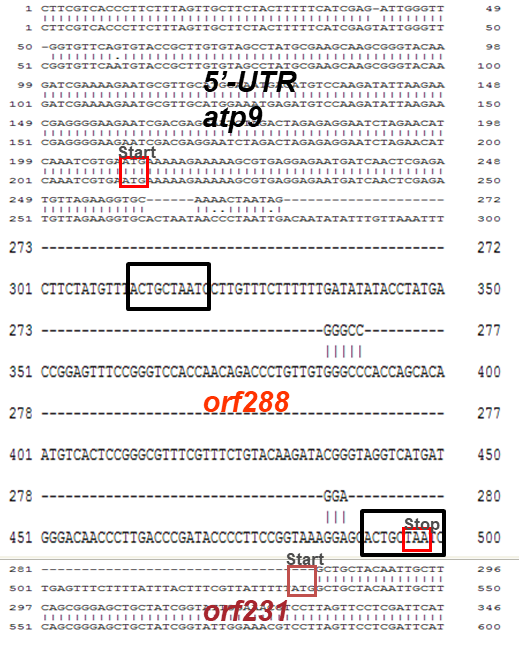


Figure S2: Organization and origin of *orf288* and *orf231* on sequence level. Red boxes - Start codons, dark red box - Stop codon, black box – direct repeat sequence ACTGCTAATC.

>orf231 (mtDNA)

ATGGCTGCTACAATTGCTTCAGCGGGAGCTGCTATCGGTATTGGAAACGTCCTTAGTTCCTCGATTCATTCCGTGGCTCGGAATCCATCATTGGCTAAACAATCATTTGGTTATGCCATTTTGGGCTTTGCTCTAACCGAAGCTATTGCATCGTTTGCCCCAATGATGGCCTTTCTGATCTCATCCGTATTCCGATCAAAGAATCAAAGAAAGAAGGTTGAAGTTTCATAA

>orf231 **(editing sites present in *atp9* are marked in red, mtDNA)**

ATGGCTGCTACAATTGCTT**C**AGCGGGAGCTGCTATCGGTATTGGAAACGT**CC**TTAGTTCCT**C**GATTCATTCCGTGGCTCGGAATCCATCATTGGCTAAACAAT**C**ATTTGGTTATGCCATTTTGGGCTTTGCTCTAACCGAAGCTATTGCAT**C**GTTTGCCC**C**AATGATGGCCTTT**C**TGATCT**C**AT**C**CGTATTC**C**GATCAAAGAATCAAAGAAAGAAGGTTGAAGTTTCATAA

>orf231 **(edited sequence, cDNA)**

ATGGCTGCTACAATTGCTT**T**AGCGGGAGCTGCTATCGGTATTGGAAACGT**TT**TTAGTTCCT**T**GATTCATTCCGTGGCTCGGAATCCATCATTGGCTAAACAAT**T**ATTTGGTTATGCCATTTTGGGCTTTGCTCTAACCGAAGCTATTGCAT**T**GTTTGCCC**T**AATGATGGCCTTT**T**TGATCT**T**AT**T**CGTATTC**T**GATCAAAGAATCAAAGAAAGAAGGTTGAAGTTTCATAA

>orf288 (mtDNA)

ATGAAAAAGAAAAAGCGTGAGGAGAATGATCAACTCGAGATGTTAGAAGGTGCACTAATAACCCTAATTGACAATATATTTGTTAAATTTCTTCTATGTTTACTGCTAATCCTTGTTTCTTTTTTGATATATACCTATGACCGGAGTTTCCGGGTCCACCAACAGACCCTGTTGTGGGCCCACCAGCACAATGTCACTCCGGGCGTTTCGTTTCTGTACAAGATACGGGTAGGTCATGATGGGACAACCCTTGACCCGATACCCCTTCCGGTAAAGGAGCACTGCTAA

>orf288 (cDNA)

ATGAAAAAGAAAAAGCGTGAGGAGAATGATCAACTCGAGATGTTAGAAGGTGCACTAATAACCCTAATTGACAATATATTTGTTAAATTTCTTCTATGTTTACTGCTAATCCTTGTTTCTTTTTTGATATATACCTATGACCGGAGTTTCCGGGTCCACCAACAGACCCTGTTGTGGGCCCACCAGCACAATGTCACTCCGGGCGTTTCGTTTCTGTACAAGATACGGGTAGGTCATGATGGGACAACCCTTGACCCGATACCCCTTCCGGTAAAGGAGCACTGCTAA

Figure S3: RNA-editing analysis of *orf288* and *orf231*. Genomic and cDNA sequences are shown in comparison. Editing sites in *atp9* are marked in red.

A

B

Figure S4: Sequences of the two PCR bands by PET2spec_for/PET2spec_rev in CMS GIG1. A. *orf288* (light yellow) and *orf231* (light blue) in the 743-bp-fragment, B. *atp9* (yellow) in the 491-bp-fragment, primer sequences in grey
